# Effect of skin tone on the accuracy of the estimation of arterial oxygen saturation by pulse oximetry: a systematic review

**DOI:** 10.1016/j.bja.2024.01.023

**Published:** 2024-02-17

**Authors:** Daniel Martin, Chris Johns, Lexy Sorrell, Eugene Healy, Mandeep Phull, Segun Olusanya, Mark Peters, Jeremy Fabes

**Affiliations:** 1 Peninsula Medical School, University of Plymouth, John Bull Building, Plymouth, UK; 2 Intensive Care Unit, University Hospitals Plymouth, Plymouth, UK; 3 Library & Digital Support, University of Plymouth, Drake Circus, Plymouth, UK; 4 Dermatopharmacology, Clinical and Experimental Sciences, Faculty of Medicine, University of Southampton, Southampton, UK; 5 Dermatology, University Hospital Southampton NHS Foundation Trust, Southampton, UK; 6 Barking, Havering and Redbridge University Trust, Romford, UK; 7 William Harvey Research Institute, Queen Mary University London, London, UK; 8 St Bartholomew's Hospital, London, UK; 9 Paediatric Intensive Care Unit, Great Ormond Street Hospital for Children NHS Foundation Trust and NIHR Biomedical Research Centre, London, UK; 10 University College London Great Ormond St Institute of Child Health, London, UK; 11 Anaesthetic Department, University Hospitals Plymouth, Plymouth, UK

**Keywords:** ethnicity, hypoxaemia, oximetry, racial bias, review, skin tones, skin type

## Abstract

**Background:**

Pulse oximetry-derived oxygen saturation (SpO_2_) is an estimate of true arterial oxygen saturation (SaO_2_). The aim of this review was to evaluate available evidence determining the effect of skin tone on the ability of pulse oximeters to accurately estimate SaO_2_.

**Methods:**

Published literature was screened to identify clinical and non-clinical studies enrolling adults and children when SpO_2_ was compared with a paired co-oximetry SaO_2_ value. We searched literature databases from their inception to March 20, 2023. Risk of bias (RoB) was assessed using the QUADAS-2 tool. Certainty of assessment was evaluated using the GRADE tool.

**Results:**

Forty-four studies were selected reporting on at least 222 644 participants (6121 of whom were children) and 733 722 paired SpO_2_–SaO_2_ measurements. Methodologies included laboratory studies, prospective clinical, and retrospective clinical studies. A high RoB was detected in 64% of studies and there was considerable heterogeneity in study design, data analysis, and reporting metrics. Only 11 (25%) studies measured skin tone in 2353 (1.1%) participants; the remainder reported participant ethnicity: 68 930 (31.0%) participants were of non-White ethnicity or had non-light skin tones. The majority of studies reported overestimation of SaO_2_ by pulse oximetry in participants with darker skin tones or from ethnicities assumed to have darker skin tones. Several studies reported no inaccuracy related to skin tone. Meta-analysis of the data was not possible.

**Conclusions:**

Pulse oximetry can overestimate true SaO_2_ in people with darker skin tones. The clinical relevance of this bias remains unclear, but its magnitude is likely to be greater when SaO_2_ is lower.

**Systematic review protocol:**

International Prospective Register of Systematic Reviews (PROSPERO): CRD42023390723.

**Editor's key points**

• It has been suspected that pulse oximeters overestimate arterial oxygen saturation (SaO_2_) in people with darker skin tones. Conflicting evidence has made it difficult to confirm or quantify this potential bias.
• This systematic review included all relevant studies to date and concluded that most studies reported an overestimation of SaO_2_ by pulse oximetry and this was greatest in people with the darkest skin tones.
• The magnitude of the bias was difficult to determine, and its clinical consequences are yet to be elucidated.

Pulse oximetry provides a noninvasive approximation of the amount of oxygen in arterial blood. The value this device produces is termed peripheral arterial haemoglobin oxygen saturation (SpO_2_), which is an estimation of true arterial haemoglobin oxygen saturation (SaO_2_). Although haemoglobin is the primary source of absorption of the two wavelengths used in pulse oximetry, melanin, a pigment found in skin, can also absorb these wavelengths.^1^ Traditional understanding of pulse oximetry was that skin pigmentation should not influence the accuracy of pulse oximeters because melanin within the skin absorbs a constant, non-pulsatile fraction of the transmitted light. As only the pulsatile fraction of the detected light signal is used in the pulse oximeter algorithm to determine SpO_2_, the nonpulsatile light absorption by melanin should be irrelevant. However, early data from small laboratory and clinical studies suggested that this may not be the case^2^^,^^3^ and after a landmark report published in 2020,^4^ a number of retrospective studies have suggested that SpO_2_ overestimates SaO_2_ in individuals with darker skin tones.

The recent COVID-19 pandemic led to a focus on specific SpO_2_ thresholds for treatment initiation, reminding us of the importance of medical device accuracy in acutely unwell patients. There has been considerable debate about the effect of skin tone on pulse oximeter accuracy,^5^, ^6^, ^7^ and the topic has recently been summarised in this journal.^8^ This has triggered concerns about the limitations of pulse oximetry in certain circumstances and the regulatory processes that govern these devices.^9^ It is important to remain abreast of new evidence in this field and consider it systematically, particularly as not all studies have reached similar conclusions. The purpose of this review was to gather and synthesise all evidence to date that describes original research evaluating the effect of ethnicity, skin tone, or both on the accuracy of pulse oximetry to estimate SaO_2_. In doing so, the aim was to determine whether there is a robust body of evidence to support the suggestion that pulse oximetry overestimates SaO_2_ in people with darker skin tone. If confirmed, the importance of this bias would be that overestimation of true oxygenation might lead to a failure to recognise clinically important hypoxaemia, resulting in delayed treatment, harm, or both.

## Methods

This systematic review was reported in accordance with the international Preferred Reporting Items for Systematic Reviews and Meta-Analyses (PRISMA) 2020 updated guidelines^10^ and was registered with the International Prospective Register of Systematic Reviews (PROSPERO): CRD42023390723.

### Eligibility criteria

Inclusion criteria comprised: (1) studies that enrolled human participants (adults or children) to conduct original research in which pulse oximetry readings were compared with a reference standard to enable an evaluation of accuracy; (2) the index test was pulse oximetry-derived SpO_2_; (3) the reference standard (comparator) was arterial blood gas (ABG) co-oximetry measured SaO_2_; (4) prospective or retrospective design; (5) laboratory experiments that enrolled healthy volunteers; (6) clinical studies that enrolled patients; and (7) pulse oximeter accuracy was determined according to ethnicity or skin tone.

Exclusion criteria comprised: (1) studies of animals; (2) studies not including human participants (e.g. the use of experimental phantoms); and (3) no acceptable reference standard was used.

The eligibility of studies was not restricted by geography or setting.

### Information sources

We searched MEDLINE (Ovid), Embase (Ovid), CINAHL (EBSCO), and Web of Science. Studies published since the inception of each database until the search date (March 20, 2023) were included. Only studies which were obtainable in English were included. The reference list of articles selected for full-text review along with review articles identified by the search were screened for additional studies that had not appeared in the search results.

### Search strategy

The search strategy was developed following the PICO (population, intervention, comparator, outcome) search formula and was peer reviewed using the Peer Review of Electronic Search Strategy (PRESS) guideline.^11^ The strategy incorporated the BAME search hedge produced by Library and Knowledge Service for NHS Ambulance Services in England (https://ambulance.libguides.com/searchblock/BAME) for each respective source where available. Search terms incorporated proximity syntax and all fields were searched to ensure widest recall. No limits or filters were applied. The full search strategies are detailed in the Supplementary material.

### Search strategy selection

Titles and abstracts of potentially eligible studies were screened by two reviewers independently. The full text of all studies that either reviewer identified as potentially eligible was retrieved and the same two reviewers independently screened these against inclusion and exclusion criteria, with any disagreements resolved through consensus or by a third reviewer.

### Data collection

Data were extracted in a standardised manner by the first reviewer, randomly checked by the second reviewer, and discrepancies resolved by a third reviewer.

### Data items

Supplementary Tables S2–S4 show the structure of the tool used to extract data from reports. Information relating to the measurement of participant skin tone, ethnicity, or both was recorded. The primary outcome was any measure of accuracy of the tested pulse oximeter(s). Accuracy can be evaluated by Bland and Altman plots^12^ to calculate bias (systematic error, the mean difference between SpO_2_ and SaO_2_), precision (random error, the standard deviation of mean difference), and overall accuracy (root mean square error), which combines bias and precision. Another commonly used patient-focused metric is termed ‘occult hypoxaemia’; when true hypoxaemia (e.g. an SaO_2_ <88%) goes undetected by pulse oximetry SpO_2_ as a result of overestimation of SaO_2_ (e.g. an SpO_2_ of 92–96%).

Other information extracted included study setting (laboratory or clinical), type of participants (healthy volunteers, patients, adults, children), make and model of pulse oximeter (if known), and type of reference standard.

### Risk of bias assessment

Risk of bias (RoB) was assessed using the criteria set out in the QUADAS-2 tool for evaluation of diagnostic studies, with the RoB being classified a low, high, or unclear for each assessment domain.^13^

### Synthesis methods

Statistical analyses, including meta-analysis, were not undertaken.

### Certainty assessment

The quality of the selected evidence was evaluated using the GRADE criteria (https://gdt.gradepro.org/app/handbook/handbook.html) taking into consideration factors said to be of importance in the absence of a single estimate of effect.^14^ These were then discussed amongst all authors to determine the final rating.

### Terminology

Language is constantly evolving, and it is essential to use the most accurate and acceptable terms at the time of writing. Whether studies described skin colour, pigmentation, or tone, we chose to always report using the term skin tone. Whether studies described race or ethnicity, we chose to always report using the term ethnicity. In the latter case, this was primarily based on guidance produced by the UK Cabinet Office, but appreciate terminology can vary from country to country.^15^

## Results

### Study selection

**Fig 1:**
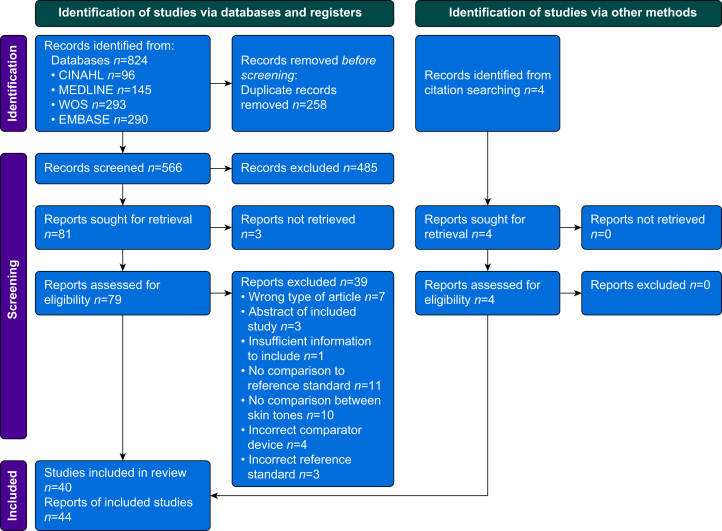
PRISMA diagram describing the identification, screening, and inclusion of reports for the systematic review.^10^

The initial search yielded 824 results of which 258 were duplicates, leaving 566 potential studies to screen. Eighty-one studies were identified from abstracts as being potentially eligible, two of which had no full text available, leaving 79 full-text manuscripts to evaluate. Of the 79 full-text manuscripts, 40 were eligible for the review; the reasons for excluding the other 39 are detailed in Figure 1. Additionally, four eligible studies were identified from the references of screened manuscripts and added to the review, making the total number of included studies 44.

### Study characteristics

The 44 eligible studies included at least 222 644 participants (one study did not report participant numbers^16^) and at least 733 722 paired SpO_2_–SaO_2_ measurements (not all studies reported these data). Summary details are shown in Table 1. Amongst the participants, 68 930 (31.0%) were of non-White ethnicity or had non-light skin tones and 6121 (2.7%) were children. A total of 15 studies undertook a measurement of skin tone, and the remaining studies only reported participant ethnicity. After reviewing the design of the eligible studies, they were divided into either healthy volunteer (*n*=8), prospective clinical (*n*=18), or retrospective clinical (*n*=18) studies. The methodology between these categories was markedly different, so results in this review were organised by study design for clarity.

**Table 1:** Summary characteristics of the included studies. ∗For those studies reporting those data.

Item	Summary statistic
**Overall**	
Total number of participants	>222 644∗
Total number of paired measurements	>733 722∗
Average number of paired measurements per participant	3.3∗
Study design	
• Prospective healthy volunteer	8 (18.6%)
• Prospective clinical	18 (40.9%)
• Retrospective clinical	18 (41.8%)
Adult participants	216 551 (97.2%)
Child participants	6121 (2.7%)
Participants for which skin tone was reported	2353 (1.1%)
Participants of non-White ethnicity or with darker skin tones	68 930 (31.0%)
**Prospective healthy volunteers**	
Total number of participants	438
Total number of paired measurements	18 892∗
Average number of paired measurements per participant	60.2∗
Adult participants	438 (100%)
Child participants	0
Participants for which skin tone was reported	270 (61.6%)
Participants of non-White ethnicity or with darker skin tones	257 (58.7%)
**Prospective clinical**	
Total number of participants	2170
Total number of paired measurements	5234
Average number of paired measurements per participant	4.3
Adult participants	1827 (84.2%)
Child participants	343 (19.4%)
Participants for which skin tone was reported	1853 (85.4%)
Participants of non-White ethnicity or with darker skin tones	780 (35.9%)
**Retrospective clinical**	
Total number of participants	220 036∗
Total number of paired measurements	709 587∗
Average number of paired measurements per participant	3.2
Adults	214 258 (97.4%)
Children	5778 (2.6%)
Skin tone reported	0
Non-White ethnicity/darker skin tones	67 893 (30.9%)

#### Healthy volunteer studies

Eight laboratory studies, published between 1976 and 2023, were reviewed comprising a total of 438 healthy adult volunteers (Table 1). Two studies (*n*=221) were industry-funded retrospective analyses of historic laboratory data and it was not possible to determine if data from studies already included in the review were contained within these.^17^^,^^18^ All of the studies involved participants breathing ≤21% oxygen in order to evaluate the effect of lowering blood oxygenation. A range of pulse oximeters were evaluated in this category; many early devices are now obsolete including the multiple wavelength Hewlett-Packard 47201A. Evaluation of skin tone only occurred in three studies.^18^, ^19^, ^20^ The proportion of participants with darker skin tones or of non-White ethnicities was particularly low in earlier studies (Supplementary Table S2). One study comprised 100% Black participants.^2^

#### Prospective clinical studies

The 18 prospective clinical studies (Table 1) were published between 1985 and 2022; they included a total of 2170 patients and at least 5243 paired SpO_2_–SaO_2_ measurements. A range of pulse oximeters were evaluated and similar to the healthy volunteer studies, many from earlier reports are now obsolete. The reference standard was ABG co-oximetry in all studies. Participants included hospitalised adults,^21^, ^22^, ^23^ adult outpatients,^3^^,^^21^^,^^23^, ^24^, ^25^ adults admitted to emergency departments (EDs),^26^ adults on intensive care units (ICUs) or high-dependency units (HDUs),^23^^,^^27^, ^28^, ^29^, ^30^, ^31^ adults following cardiac surgery,^32^ children on ICUs,^33^, ^34^, ^35^, ^36^ and children with cyanotic heart disease.^37^ A method to measure skin tone was used in 11 of the 18 studies.^3^^,^^22^, ^23^, ^24^^,^^26^^,^^28^, ^29^, ^30^^,^^33^^,^^36^^,^^37^ The proportion of participants with darker skin tones or of non-White ethnicities largely reflected the local population characteristics. One study enrolled only participants with dark skin tones.^28^

#### Retrospective clinical studies

More retrospective clinical studies were published between December 2020 and March 2023 than there were prospective studies from 1970 to 2023 (Table 1). They included at least 220 036 participants and 709 587 paired measurements. These studies used previously collected data derived from hospital records, extracting paired SpO_2_–SaO_2_ measurements. The gap between measuring SpO_2_ and SaO_2_ ranged from <1 min^38^ to 30 min.^39^ Most studies reported occult hypoxaemia rather than bias; however, at least five different definitions for occult hypoxaemia were detected amongst the selected reports. Reporting included the proportion of patients with occult hypoxaemia at some point and the proportion of paired readings with occult hypoxaemia, whereas some studies included more than one paired reading per patient. The denominator for the calculation of the percentage of occult hypoxaemia varied between studies and included the total number of patients/readings and the number of SpO_2_ readings without hypoxia (1 – the negative predictive value). Three studies^40^, ^41^, ^42^ reported the percentage of occult hypoxaemia out of true hypoxaemia (1 – sensitivity); this value is not influenced by the prevalence of hypoxaemia. Only three studies reported the make of pulse oximeter evaluated^42^, ^43^, ^44^ and all studies used ABG-derived SaO_2_ as the reference standard. All of the studies used inpatient data; settings included adult ICUs,^4^^,^^38^^,^^41^^,^^43^^,^^45^, ^46^, ^47^, ^48^ adult wards,^4^^,^^16^^,^^39^^,^^40^^,^^49^ adults in an ED,^50^ adults undergoing anaesthesia,^44^ paediatric wards,^51^^,^^52^ paediatric cardiac catheterisation,^53^ and prematurely born infants.^42^ All the studies analysed mixed ethnicity populations that reflected the regional population characteristics. None of the studies measured skin tone, instead self-reported ethnicity was used as a surrogate for skin tone when evaluating pulse oximeter accuracy.

### Risk of bias

Risk of bias for each study is summarised in Supplementary Table S1. High RoB was identified in 28/44 (64%) of the studies. In general, the RoB varied in nature between the three categories of study. Common themes included non randomised or nonconsecutive selection of participants in healthy volunteer studies; unique clinical scenarios in the prospective clinical studies (hypothermia, during exercise, in newborns); and a varied time delay between the SpO_2_ and SaO_2_ measurements in retrospective clinical studies.

### Results of individual studies

#### Healthy volunteer studies

Data from these studies are found in Supplementary Table S2. The first two studies in this group used correlation to compare SpO_2_ with SaO_2_ and reported no skin tone-related inaccuracy.^54^^,^^55^ All the remaining studies presented the bias (mean [standard deviation] difference) between SpO_2_ and SaO_2_ values. For patients with dark skin tones, four studies reported overestimation of SaO_2_ by pulse oximetry,^2^^,^^19^^,^^56^ one reported underestimation,^17^ and one (a conference abstract) reported increased errors.^18^ The magnitude of bias varied between studies, particularly in relation to SaO_2_; lower SaO_2_ was associated with a greater degree of overestimation bias. Bias of up to 10.4 (6.8)% was reported in profoundly hypoxaemic participants (SaO_2_ approximately 75%) using an old device no longer in service (Biox Ohmeda IIA ear probe [Ohmeda, Boulder, CO, USA]).^54^ The bias was smaller in less dated models (combined data from Nonin Onyx [Nonin Inc, Plymouth, MA, USA], Novametrix 513 [Novametrix Inc, Wallingford, CT, USA], and Nellcor N-595 [Nellcor Inc, Hayward, CA, USA]): 0.17 (1.29)% at an SaO_2_ of 90–100%, 0.93 (1.64)% at an SaO_2_ of 80–90%, 2.01 (2.30)% at an SaO_2_ of 70–80%, and 3.56 (2.45)% at an SaO_2_ of 60–70%.^56^ Similarly, a bias of 2.4–3.6% at an SaO_2_ range of 70–80% for Nellcor N-595 and Nonin 9700 pulse oximeters with adhesive probes and 4.5–4.9% was reported at an SaO_2_ of 60% and 70%.^19^

Three studies^2^^,^^54^^,^^55^ evaluated the Hewlett-Packard 47201A [Hewlett-Packard, Waltham, MA, USA], which is not strictly a pulse oximeter as it uses multiple wavelengths of light.^57^ One of these three studies presented bias data and reported a consistent underestimation of true SaO_2_ in a cohort of 33 Black participants with a maximum bias of –7.1 (9.0)%.^20^^,^^24^^,^^25^^,^^27^^,^^31^^,^^33^^,^^35^^,^^36^

The two studies summarising collated data from numerous laboratory studies and analysed by manufacturers (Medtronic [Dublin, Ireland] and Masimo [Irvine, CA, USA]) failed to demonstrate a difference in bias related to skin tone.^17^^,^^18^

#### Prospective clinical studies

Details of these studies are found in Supplementary Table S3. All 18 studies used ABG-derived SaO_2_ as the reference standard, but there was no standardised statistical method used to report accuracy.

Nine (50%) of the 18 studies reported no inaccuracy related to skin tone,^21^^,^^25^^,^^26^^,^^28^^,^^32^^,^^34^^,^^36^^,^^37^ although one of these studies did note that pulse oximetry functioned poorly more often in those with darker skin tones.^26^ The largest of the studies reporting no inaccuracy attributable to skin tone enrolled 400 hospitalised participants from hospitals in Australia and New Zealand, and tested several different pulse oximeters.^23^ Although the investigators assessed skin tone using the Fitzpatrick scale, there was only one participant in the group defined as having dark skin (Fitzpatrick V and VI), so they could only compare light (Fitzpatrick I and II) to medium (Fitzpatrick III and IV) skin tones. Another large study reporting no inaccuracy enrolled 295 adults admitted to an ED in the USA.^26^ All pulse oximetry measurements were performed using the Nellcor D-25 [Nellcor Inc, Hayward, CA, USA] and the investigators assessed skin tone using a colour chart system (the Munsell colour system) which was then categorised into light, intermediate, or dark. Although no difference was reported in bias or precision between the skin tone groups, investigators noted that suboptimal pulse oximeter function (unstable signal) occurred in 11% of the light skin tone group, 11% of the intermediate skin tone group, and 32% of the dark skin tone group (*P*=0.003). In another relatively large study that enrolled 100 critically ill patients with dark skin tones, the investigators used reflectance spectrophotometry to quantify skin tone objectively.^28^ Three different pulse oximeters were evaluated and although there was variation in their accuracy, they all demonstrated good precision and bias values that were within the manufacturers' quoted ranges. However, most of the SaO_2_ values in this study were above 95%. A relatively large study evaluated three pulse oximeters in 101 adult outpatients with chronic respiratory disease undertaking an exercise test.^25^ Only 5% of participants had moderately dark skin tones and the method of determining this was not stated in the manuscript. All the remaining studies reporting no inaccuracy had 50 or fewer participants. Of note, three of these smaller studies were conducted in cohorts of hospitalised children in the USA.^34^^,^^36^^,^^37^ Two of these were published within the last 10 yr, using equipment likely to still be available to clinicians.^36^^,^^37^

Amongst those reporting skin tone-related inaccuracy, the largest study was conducted relatively recently (in 2018) using pulse oximeters currently in use (manufactured by Masimo and Philips [Eindhoven, the Netherlands]).^29^ Investigators recruited 394 critically ill adults in Australia and New Zealand of whom 17.1% were from an ethnicity likely to have darker skin tones. The Fitzpatrick scale was used to measure skin tone and darker skin tone was associated with an overestimation of SaO_2_ by pulse oximetry. However, the magnitude of this effect was reported as being small (2.4 [95% confidence interval 1.2–3.6]% for light *vs* dark skin tones). A study conducted in 225 hypoxaemic children admitted to hospital reported a lower likelihood of bias in African–American participants (*n*=20) compared with other ethnicities using Masimo and Nellcor pulse oximeters.^35^ It was the only study in this review to report lower bias in a patient cohort with darker skin tones. In the other studies, inaccuracy was detected in adults with respiratory disease undertaking an exercise test,^3^^,^^24^ infants in a neonatal unit,^33^ mechanically ventilated adults post cardiac surgery,^32^ hospitalised adult patients,^22^ and critically ill adults on an ICU.^27^^,^^30^^,^^31^ All reported greater overestimation of SaO_2_ by pulse oximetry in participants with darker skin tones.

#### Retrospective clinical studies

**Fig 2:**
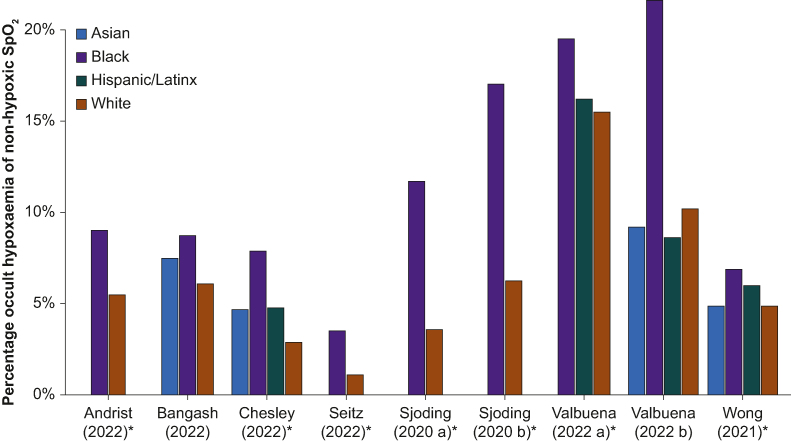
Frequency of occult hypoxaemia in paired SpO_2_–SaO_2_ measurements amongst non-hypoxaemic SpO_2_ readings. Bar chart of the retrospective clinical studies displaying the percentage of readings with occult hypoxaemia out of readings/patients with non-hypoxaemic SpO_2_ (definitions for each study provided in Supplementary Table S5). The percentage reflects one minus the negative predictive value for the detection of hypoxaemia by SpO_2_, therefore the results within each study are affected by the (unknown) prevalence of true hypoxaemia, and comparison cannot be drawn between studies (comparison within studies requires the assumption of equal prevalence between groups). ∗Studies including multiple readings per patient. a and b describe separate cohorts within the same manuscript.

**Fig 3:**
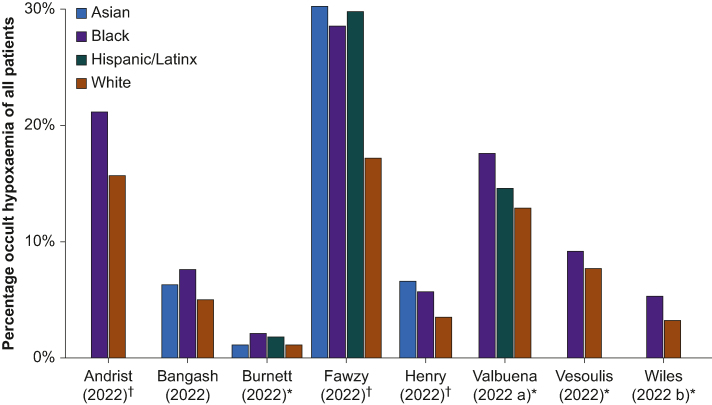
Frequency of occult hypoxaemia amongst patients. Bar chart of the retrospective clinical studies displaying the percentage of readings with occult hypoxaemia out of all readings/patients (definitions for each study provided in Supplementary Table S5). The percentages are affected by the (unknown) prevalence of true hypoxaemia, and comparison cannot be drawn between studies (comparison within studies requires the assumption of equal prevalence between groups). ∗Studies including multiple readings per patient. ^†^Studies reporting occult hypoxaemia from at least one reading per patient.

**Fig 4:**
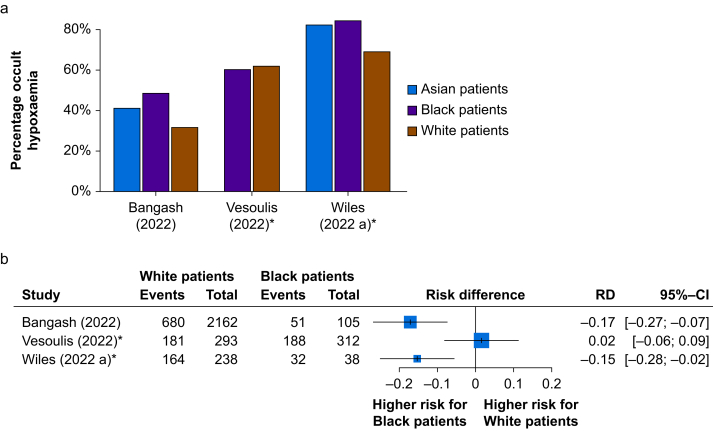
(a) Occult hypoxaemia in those patients with true hypoxaemia. Bar chart of the retrospective clinical studies displaying the percentage of readings with occult hypoxaemia out of readings/patients with true hypoxaemia (definitions for each study provided in Supplementary Table S5). The percentage reflects one minus the sensitivity for the detection of hypoxaemia by SpO_2_. (b) Forest plot of occult hypoxaemia in White and Black patients with true hypoxaemia. Forest plot of the retrospective clinical studies displaying the risk difference (RD) between reading from White and Black patients with occult hypoxaemia out of readings/patients with true hypoxaemia. The percentage reflects one minus the sensitivity for the detection of hypoxaemia by SpO_2_. ∗Studies including multiple readings per patient. CI, confidence interval.

Details of these studies are shown in Supplementary Table S4. Most studies compared the frequency of occult hypoxaemia in different ethnic groups. Comparing findings between studies was complicated by investigators using different methods to analyse and report data such that the denominator for occult hypoxemia varied between them. Fig 2, Fig 3, Fig 4 display the summary data from studies reporting occult hypoxaemia. A forest plot is presented (Fig 4a) to display the risk difference of SpO_2_ not detecting hypoxaemia in hypoxaemic White and Black patients. However, forest plots for the risk difference of occult hypoxaemia out of all patients and those with normal SpO_2_ readings (displayed in Fig 2, Fig 3) are not presented as the prevalence of hypoxaemia is unknown and might not be equal between studies or between White and Black patients. The definition of occult hypoxaemia varied between reports and this is outlined in Supplementary Table S2. Only one relatively small retrospective study that analysed data from 194 adult patients with COVID-19 pneumonitis (28% of whom were of non-White ethnicity) detected no significant effect of ethnicity on the accuracy of pulse oximetry.^41^ All the remaining 17 studies reported some degree of pulse oximetry inaccuracy related to self-reported ethnicity, with a tendency for pulse oximetry to overestimate SaO_2_ in patients whose ethnicity would be associated with darker skin tones. The magnitude of difference between the frequency of occult hypoxaemia between ethnic groups tended to be greatest between White and Black participants and least between White and Asian participants, when the latter was reported (Fig 2, Fig 3, Fig 4). When comparing the frequency between White and Black patients, occult hypoxaemia was reported as being up to three times greater amongst Black patients.^4^^,^^48^ For Asian *vs* White patients, the difference in occult hypoxaemia frequency was not greater than two-fold,^38^^,^^44^^,^^49^ whereas one study showed a lower frequency of occult hypoxaemia amongst Asian patients^43^ and three showed no difference.^16^^,^^41^^,^^45^ A number of studies reported data from Hispanic or Latino patients, reporting a higher frequency,^44^^,^^45^^,^^49^ or no difference to^16^ White participants. The only study that reported data from indigenous American participants showed them to have a significantly higher frequency of occult hypoxaemia than White participants.^38^

Given the timing of the publications in this category, numerous studies reported data from patients with COVID-19 and findings from these studies did not appear to be different from those patients who did not have COVID-19. Three studies used data from children and in all of these studies, greater pulse oximetry inaccuracy was observed in Black children.^51^, ^52^, ^53^

### Studies in which skin tone was measured and reported

Of the 13 studies in which an assessment of skin tone was undertaken, eight (61.5%) of them reported inaccuracies related to skin tone. A variety of methods were used to measure skin tone including visual inspection and grading, comparison to colour charts, and spectrophotometry. Of those that did not report any inaccuracies, one only recruited participants with dark skin tones (without a light skin tone comparator) and evaluated relatively high SaO_2_ readings (mainly >94%).^28^ In addition, one was a relatively large prospective study of 295 adults which observed that pulse oximetry functioned poorly more often in those with dark skin (but no bias was reported),^26^ one only compared light *vs* medium skin tones (not dark skin tones),^23^ and the remaining two were very small studies of children.^36^^,^^37^

### Data analysis

Meta-analysis was not possible in either the whole cohort of studies, or in subsets. The key reasons were heterogeneity in study design, population, pulse oximeter make, and data reported. The lack of required data available in published reports was also significant. Further details are outline in the Supplementary material.

### Certainty of evidence

The certainly of the findings in this review was rated as moderate, that is, the true effect is likely to be close to the estimate of the effect, but it is possible they are substantially different.

## Discussion

Most studies (30/44, 68%) in this review reported that pulse oximetry was more likely to overestimate SaO_2_ in participants with darker skin tones (i.e. greater bias) and a number of studies stated that this error was exacerbated at lower values of SaO_2_. One reported lower bias in participants with darker skin tones. The magnitude of overestimation (bias) was frequently small and determining the clinical relevance of this is difficult. Data from older studies may not be relevant as the pulse oximeters evaluated are now largely obsolete. Study design and the methodology used to evaluate accuracy varied widely across the studies in this review as did the approach to data analysis and reporting. The technical reasons for detected inaccuracies remain unclear but may be related to the light source spectra used by pulse oximeters and the diversity of skin tone in participants enrolled into original pulse oximeter calibration studies.^58^

The results of this review are in line with other systematic reviews, one of which was published in 2022 and reported on 32 studies including only 6505 participants.^59^ The authors managed to conduct a meta-analysis of assorted outcome and concluded that pulse oximetry overestimated true SaO_2_ in people with the darkest skin tones (pooled mean bias 1.11%; 95% confidence interval 0.29–1.93%). Understanding the validity of the meta-analysis is difficult given the heterogenous nature of the data entered into it. The second review was also published in 2022 and included 41 articles, such as letters, review articles, and case reports.^60^ The authors performed a bibliometric analysis and concluded that although some older studies did not find any bias related to darker skin tones, more recent studies tended to show that pulse oximeters have limitations for patients with dark skin tone, particularly when SaO_2_ is low.

Retrospective studies contributed the greatest proportion of participants and paired measurements to this review and all but one of them reported inaccuracies related to ethnicity (Supplementary Table S4; Fig 2, Fig 3, Fig 4). Although findings from retrospective studies come with the caveat of interpretation from large databases that were not designed with this analysis in mind, they provide an important body of evidence that is difficult to ignore. All of these studies were conducted in high-income countries where the majority of the population have light skin tones. The major finding in this cohort of studies relates to the incidence of occult hypoxaemia (an important clinical metric) with an incidence of up to 21.5% in Black patients. Most studies reported an occult hypoxaemia incidence 25–75% higher in Black compared with White patients, with other ethnic groups having a 25–50% higher incidence. Furthermore, error and bias in readings in Black compared with White patients worsened with greater degrees of hypoxaemia, especially in the clinically important SaO_2_ range of 85–88% where deterioration can be rapid.

Only nine studies exclusively enrolled children, with a total of 6121 participants.^33^, ^34^, ^35^, ^36^, ^37^^,^^42^^,^^51^, ^52^, ^53^ There were no healthy volunteer studies of children. Six of the nine studies reported skin tone-related inaccuracies.^33^^,^^35^^,^^42^^,^^51^, ^52^, ^53^ Given the low overall number of children, it is difficult to draw clear conclusions, but inaccuracies were commonly reported.

### Limitations of the evidence

As with all systematic reviews, the findings are dependent on the quality of the original research. Significant numbers of included studies were performed some time ago with associated limitations on reporting standards. Particularly in the prospective group of studies, the number of included patients were low which creates significant uncertainty in the findings. A number of prospective studies^21^^,^^22^^,^^25^^,^^31^^,^^32^^,^^54^^,^^55^ used correlation to evaluate accuracy, which has significant methodological limitations, so the results of these studies should be viewed with caution. However, the number of included studies with comparable findings and conclusions suggests that despite this heterogeneity the overall conclusion is well-founded.

A wide range of pulse oximeter types were used in these studies and a large proportion of the earlier studies used pulse oximeters no longer in clinical use which may limit the transferability of these findings to modern practice. From this review, it was not possible to determine whether pulse oximeters have become more or less accurate over time. Although all prospective studies reported the manufacturer and model of pulse oximeter evaluated, the majority of post-2020 retrospective clinical studies did not. Given that most of the retrospective studies were extracted from recently constructed hospital databases using modern equipment currently in use, we can conclude that skin tone-related inaccuracies are not a historic phenomenon. Additionally, some studies reported markedly different accuracy between pulse oximeters tested within the same study, for example, the relative study by Ebmeier and colleagues,^29^ but it was outside of the remit of this review to provide a detailed analysis of performance characteristics of individual devices.

The broad range of outcome metrics described in the studies with variable criteria and definitions means that some caution must be applied. Similarly, a wide range of different devices for measuring SaO_2_ with ABG co-oximetry were used. This heterogeneity contributed to meta-analysis not being possible.

Perhaps the most important limitation in most studies was that skin tone was either not measured or was measured in a subjective manner, including the use of colour charts. Although certain ethnicities are associated with lighter or darker skin tones, a broad distribution of skin tones can exist within them. Measuring skin tone can be challenging and amongst studies in this review, eight used colour charts.^20^^,^^24^^,^^26^^,^^29^^,^^30^^,^^36^^,^^37^ The Fitzpatrick classification is a commonly used tool but fails to provide valid results in 40–60% of subjects^61^, ^62^, ^63^ and has recently been shown to miscategorise people as having darker skin tone when compared with objective quantification.^64^ All colour charts are prone to bias as variations in ambient lighting can influence skin colour and chart colour differently; in addition, they lack similarity with real skin colour. The ability to perceive colour accurately and similarly may differ between investigators, and racial bias can occur when an assessor from one ethnic background evaluates an individual from a different ethnic background.^65^ In only one study, an objective measure of skin tone was made using reflectance spectrophotometry.^28^ In a sizable number of studies in this review (including all retrospective clinical studies), pulse oximetry accuracy was evaluated according to a participant's self-reported ethnicity, a surrogate for skin tone. This is further complicated by the fact that the way ethnicity is defined varies between countries, therefore adding to the challenge of interpreting findings from the selected reports. The heterogeneity of skin tones within an ethnic group is only likely to reduce the magnitude of any detected inaccuracy that results from skin tone. Therefore, studies using ethnicity as a surrogate for skin tone may have underreported pulse oximeter inaccuracy.

In the retrospective studies, paired SpO_2_–SaO_2_ measurements were sought from databases with considerable variation between studies in the maximum allowed time difference between the two individual measurements. In those studies that permitted longer time windows, there is likely to be a greater margin of error as the clinical situation may have altered between the two readings.

Most of the studies were conducted in high-income countries; none appear to have included participants from low- or middle-income countries, which could be attributed to the exclusion of non-English reports. This is relevant as low- and middle-income countries often contain high proportions of inhabitants with darker skin tones and funding for healthcare may be such that pulse oximetry is the only available monitor for unwell patients.^66^^,^^67^ In addition, older equipment may be more prevalent in low- or middle-income countries. The clinical impact of inaccuracies may therefore be greater in these settings.

### Limitations of the review process

The review followed a rigorous process with pre-defined inclusion criteria, outcomes, data of interest, and methodology. Our search paradigm was developed using well-established standards and criteria, included a validated search hedge, and had a broad and comprehensive scope. This is reflected in the small number of additional studies that were identified outside the literature searches that came from screening of reference lists.

We were able to obtain full text for most of the included studies. The three studies we were unable to obtain full text were older publications that contained data likely not to be relevant to the review.

We have not performed a search of the grey literature, so we recognise that there may be other material available that we have not included in this review.

### Conclusions

Data from 44 studies evaluating the potential inaccuracy of pulse oximetry across different skin tones were extremely heterogenous and therefore impossible to combine in a way that summarising statistics could be used with confidence. However, overestimation of true SaO_2_ by pulse oximetry was reported in most studies in this review. The magnitude of this overestimation was greatest in people with the darkest skin tones and the overestimation was greater when SaO_2_ was low. The clinical ramification of this bias is unclear from these studies, but there is a potential for pulse oximetry to fail to identify hypoxaemia in patients with darker skin tones and therefore expose these patients to a greater risk of harm.

## Authors’ contributions

Study design: DM, CJ, EH, MPh, OO, MPe, JF

Screening of literature: DM, JF

Data extraction: DM

Manuscript preparation: all authors

Search strategy design: CJ

Data validation: LS, JF

Figure creation: LS

## Declarations of interest

DM, EH and OO are investigators for the ongoing NIHR-funded EXAKT study, to determine the effect of skin tone on the diagnostic accuracy of pulse oximeters (https://fundingawards.nihr.ac.uk/award/NIHR135577). DM, CJ, MPh, MPe, and EH contributed to the Equity in medical devices independent review (https://www.gov.uk/government/groups/equity-in-medical-devices-independent-review).
