# Supplementary material for: Effect of skin tone on the accuracy of the estimation of arterial oxygen saturation by pulse oximetry: a systematic review
Source: Br J Anaesth. 2024 Feb 17;132(5):945–56. doi: 10.1016/j.bja.2024.01.023 (PMC11103098; doi:10.1016/j.bja.2024.01.023)
Supplement: Multimedia component 1 [file mmc1.docx]

# Search history - Medline

Ovid MEDLINE(R) and In-Process, In-Data-Review & Other Non-Indexed Citations <1946 to March 17, 2023>

1 ("BAME" or BME or ("Black Asian" adj1 "minority ethnic") or "minority ethnic*" or "ethnic minorit*" or (racial adj5 disparit*) or (ethnic adj5 disparit*) or "people of color" or "people of colour" or POC or "racial* minorit*" or "Race Factor*" or "mixed race" or "mixed racial" or minorit* or "ethnic* group*").af. 168589

2 ("indian subcontinent" or "Black British" or bangladeshi* or bengali* or indian* or chinese or pakistani* or african* or gyps* or "irish traveller*" or roma or arab* or "afro caribbean" or "african caribbean" or afrocaribbean or "afro-caribbean" or "south asian*" or refugee* or migrant* or immigrant* or "asylum seeker*" or Jew*).af. 2354268

3 1 and 2 49640

4 *"ETHNIC GROUPS"/ or *"MINORITY GROUPS"/ or *"MINORITY HEALTH"/ or *"AFRICAN CONTINENTAL ANCESTRY GROUP"/ or *"ASIAN CONTINENTAL ANCESTRY GROUP"/ or CONTINENTAL POPULATION GROUPS/ or *"AFRICAN AMERICANS"/ or *"ASIAN AMERICANS"/ or *BLACKS/ or *"HISPANIC AMERICANS"/ or *"MEXICAN AMERICANS"/ or *"AMERICAN NATIVE CONTINENTAL ANCESTRY GROUP"/ or *"EUROPEAN CONTINENTAL ANCESTRY GROUP"/ or *"OCEANIC ANCESTRY GROUP"/ or *"RACE FACTORS"/ 161591

5 3 or 4 190111

6 (skin adj3 colo?r).af. 6673

7 (skin adj3 tone).af. 787

8 (skin adj3 pigment*).af. 15620

9 ("black skin" or "asian skin" or "white skin").af. 994

10 6 or 7 or 8 or 9 21173

11 exp skin color/ 8828

12 exp skin pigmentation/ 8828

13 10 or 11 or 12 21261

14 5 or 13 210086

15 "pulse oximet*".af. 9856

16 ("transcutaneous oximet*" or "reflectance oximet*").af. 284

17 15 or 16 10112

18 exp Oximetry/ 16570

19 17 or 18 21131

20 14 and 19 145

# Search history – Embase

Embase <1974 to 2023 March 17>

1 ("BAME" or BME or ("Black Asian" adj1 "minority ethnic") or "minority ethnic*" or "ethnic minorit*" or (racial adj5 disparit*) or (ethnic adj5 disparit*) or "people of color" or "people of colour" or POC or "racial* minorit*" or "Race Factor*" or "mixed race" or "mixed racial" or minorit* or "ethnic* group*").af. 277119

2 ("indian subcontinent" or "Black British" or bangladeshi* or bengali* or indian* or chinese or pakistani* or african* or gyps* or "irish traveller*" or roma or arab* or "afro caribbean" or "african caribbean" or afrocaribbean or "afro-caribbean" or "south asian*" or refugee* or migrant* or immigrant* or asylum seeker* or Jew*).af. 3094586

3 1 and 2 76483

4 *"MINORITY GROUP"/ or *"MINORITY HEALTH"/ or *"BLACK PERSON"/ or *"ASIAN CONTINENTAL ANCESTRY GROUP"/ or *"BRITISH ASIAN"/ or *"ETHNIC GROUP"/ or *"BLACK RACE"/ or *"BLACK POPULATION"/ or "MULTIRACIAL PERSON"/ or "BLACK PERSON"/ or *"INDIGENOUS PEOPLE"/ or *"ASIAN AMERICAN"/ or *MIGRANT/ or *"OCEANIC ANCESTRY GROUP"/ or *"ETHNIC OR RACIAL ASPECTS"/ or *"RACE DIFFERENCE"/ or *"ETHNIC DIFFERENCE"/ 80850

5 3 or 4 141017

6 (skin adj3 colo?r).af. 14927

7 (skin adj3 tone).af. 1328

8 (skin adj3 pigment*).af. 23364

9 ("black skin" or "asian skin" or "white skin").af. 1447

10 6 or 7 or 8 or 9 37383

11 exp skin color/ 24208

12 exp skin pigmentation/ 15525

13 10 or 11 or 12 37539

14 5 or 13 177615

15 "pulse oximet*".af. 29212

16 ("transcutaneous oximet*" or "reflectance oximet*").af. 408

17 exp oximetry/ 33772

18 15 or 16 or 17 40772

19 14 and 18 290

# Search history – CINAHL

CINAHL (EbscoHost)

S1 (("BAME" OR BME OR ("Black Asian" ADJ1 "minority ethnic") OR "minority ethnic*" OR "ethnic minorit*" OR (racial ADJ5 disparit*) OR (ethnic ADJ5 disparit*) OR "people of color" OR "people of colour" OR POC OR "racial* minorit*" OR "Race Factor*" OR "mixed race" OR "mixed racial" OR minorit* OR "ethnic* group*") AND ("Black British" OR bangladeshi* OR "indian subcontinent" OR bengali* OR indian* OR chinese OR pakistani* OR african* OR gyps* OR "irish traveller*" OR roma OR arab* OR "afro caribbean" OR "african caribbean" OR afrocaribbean OR "afro-caribbean" OR "south asian*" OR Refugee* OR migrant* OR Immigrant* OR asylum seeker* OR Jew*)) 21,871

S2 *"ETHNIC GROUPS"/ OR *"MINORITY GROUPS"/ OR *"MINORITY HEALTH"/ OR *"AFRICAN CONTINENTAL ANCESTRY GROUP"/ OR *"ASIAN CONTINENTAL ANCESTRY GROUP"/ OR CONTINENTAL POPULATION GROUPS/ OR *"AFRICAN AMERICANS"/ OR *"ASIAN AMERICANS"/ OR *BLACKS/ OR *"HISPANIC AMERICANS"/ OR *"MEXICAN AMERICANS"/ OR *"AMERICAN NATIVE CONTINENTAL ANCESTRY GROUP"/ OR *"EUROPEAN CONTINENTAL ANCESTRY GROUP"/ OR *"OCEANIC ANCESTRY GROUP"/ OR *"RACE FACTORS"/ 171,290

S3 S1 OR S2 173,725

S4 skin N3 colour OR skin N3 tone OR skin N3 pigment* OR "black skin" or "asian skin" or "white skin" 3,444

S5 (MH "Skin Pigmentation") 1,271

S6 S4 OR S5 3,444

S7 S3 OR S6 176,435

S8 pulse oximet* OR transcutaneous oximet* AND reflectance oximet* 5,263

S9 (MH "Pulse Oximetry") OR (MH "Pulse Oximeters") OR (MH "Oximeters+") 2,880

S10 S8 OR S9 5,344

S11 S7 AND S10 96

# Search history – Web Of Science

Web of Science Core Collection

Search #1: TS=((("BAME" OR BME OR ("Black Asian" NEAR/1 "minority ethnic") OR "minority ethnic*" OR "ethnic minorit*" OR (racial NEAR/5 disparit*) OR (ethnic NEAR/5 disparit*) OR "people of color" OR "people of colour" OR POC OR "racial* minorit*" OR "Race Factor*" OR "mixed race" OR "mixed racial" OR minorit* OR "ethnic* group*") AND ("Black British" OR bangladeshi* OR "indian subcontinent" OR bengali* OR indian* OR chinese OR pakistani* OR african* OR gyps* OR "irish traveller*" OR roma OR arab* OR "afro caribbean" OR "african caribbean" OR afrocaribbean OR "afro-caribbean" OR "south asian*" OR Refugee* OR migrant* OR Immigrant* OR asylum seeker* OR Jew*))) Results: 57191

Search #2: ALL=("ETHNIC GROUP*" OR "MINORITY GROUPS" OR "MINORITY HEALTH" OR "AFRICAN CONTINENTAL ANCESTRY GROUP" OR "ASIAN CONTINENTAL ANCESTRY GROUP*" OR "CONTINENTAL POPULATION GROUP*" OR "AFRICAN AMERICAN*" OR "ASIAN AMERICAN*" OR "BLACK*" OR "HISPANIC AMERICAN*" OR "MEXICAN AMERICAN*" OR "AMERICAN NATIVE CONTINENTAL ANCESTRY GROUP*" OR "EUROPEAN CONTINENTAL ANCESTRY GROUP*" OR "OCEANIC ANCESTRY GROUP*" OR "RACE FACTOR*") Results: 1101899

Search #3: #1 OR #2 Results: 1117645

Search #4: TS=(skin NEAR/3 colour OR skin NEAR/3 tone OR skin NEAR/3 "pigment*" OR "black skin" or "asian skin" or "white skin") Results: 24610

Search #5: #3 OR #4 Results: 1138873

Search #6: ALL=("pulse oximet*") Results: 11913

Search #7: ALL=("transcutaneous oximet*" OR "reflectance oximet*" ) Results: 317

Search #8: ALL=(oximetry) Results: 15777

Search #9: #6 OR #7 OR #8 Results: 18032

Search #10: #5 AND #9 Results: 293

**Protocol deviation**

It was highlighted by reviewers that multiple-wavelength non-invasive oximetry is not a validated technique for measuring arterial oxygenation across different skin tones. Insufficient data exist to supports its accuracy in people with darker skin tones. For that reason, the following studies initially deemed eligible for the review, were subsequently removed. All of them used a form of multiple-wavelength non-invasive oximetry as the reference standard:

Ochoa-Gutierrez 2022^1^

Chapman 1983^2^

Cahan 1990^3^

**Justification for not undertaking meta-analyses**

Most of the older prospective studies did not report any measures of accuracy that could be summarised by skin tone/ethnicity. There are three studies that could theoretically be combined in a meta-analysis as the bias (SpO_2_ – SaO_2_) was presented separately for each skin tone group.^4–6^ However, different classifications were used for skin tone (White and Black participants versus participants with light, intermediate and dark skin tones) and at least one of the studies included multiple readings per participant, so the observations were not independent.

Most of the retrospective clinical studies (except for three^7–9^) presented the proportion of occult hypoxaemia out of the total number of participants or the number of participants with SpO_2_ between 92 and 96%. The latter is equivalent to presenting the negative predictive value of SpO_2_’s ability to diagnose hypoxaemia. Both of these statistics are affected by the prevalence of true hypoxaemia, which is unreported in most studies and so these results cannot be pooled. Guides to conducting a meta-analysis of diagnostic test accuracy^10^ advise combining only sensitivity and specificity, as these values are not affected by prevalence and therefore can be pooled and compared across various scenarios. Three studies present the sensitivity of SpO_2_^7–9^, however, two of them^8, 9^ include multiple paired readings from individual participants and therefore the observations are not independent.

These reasons, combined with the heterogeneity in study design, population and pulse oximeter type have led us to conclude that a meta-analysis would not be possible at this stage.

**Supplementary table 1.** Risk of bias and applicability concerns according to the QUADAS-2 tool

|  | **RISK OF BIAS** | | | | **APPLICABILITY** | | |
| --- | --- | --- | --- | --- | --- | --- | --- |
| **AUTHOR AND YEAR** | **PATIENT SELECTION** | **INDEX TEST** | **REFERENCE STANDARD** | **FLOW AND TIMING** | **PATIENT SELECTION** | **INDEX TEST** | **REFERENCE STANDARD** |
| Saunders 1976 ^11^ | **H** | **L** | **L** | **H** | **H** | **L** | **H** |
| Mendelson 1988 ^12^ | **H** | **L** | **L** | **L** | **H** | **L** | **L** |
| Zeballos 1991 ^13^ | **H** | **L** | **L** | **L** | **H** | **L** | **L** |
| Bickler 2005 ^14^ | **H** | **L** | **L** | **L** | **H** | **L** | **L** |
| Feiner 2007 ^15^ | **H** | **L** | **L** | **L** | **H** | **L** | **L** |
| Barker 2022 ^16^ | **H** | **L** | **L** | **L** | **H** | **L** | **L** |
| Gudelunas 2022 ^17^ | **H** | **L** | **L** | **L** | **H** | **L** | **L** |
| Ajizian 2023 ^18^ | **H** | **L** | **L** | **L** | **H** | **L** | **L** |
| Wang 1985 ^19^ | **L** | **L** | **L** | **H** | **L** | **L** | **L** |
| Ries 1985 ^20^ | **L** | **L** | **L** | **H** | **H** | **L** | **L** |
| Emery 1987 ^21^ | **L** | **L** | **L** | **L** | **H** | **L** | **L** |
| Gabrielczyk 1988 ^22^ | **L** | **L** | **L** | **L** | **H** | **L** | **L** |
| Cecil 1988 ^23^ | **L** | **L** | **L** | **L** | **L** | **L** | **L** |
| Ries1989 ^24^ | **L** | **L** | **L** | **L** | **H** | **L** | **L** |
| Escourrou 1990 ^25^ | **L** | **L** | **L** | **L** | **H** | **L** | **L** |
| Lee 1993 ^26^ | **L** | **L** | **L** | **L** | **L** | **L** | **L** |
| Bothma 1996 ^27^ | **L** | **L** | **L** | **L** | **L** | **L** | **L** |
| Avant 1997 ^4^ | **L** | **L** | **L** | **L** | **L** | **L** | **L** |
| Adler 1998 ^27^ | **L** | **L** | **L** | **H** | **L** | **L** | **L** |
| Ross 2014 ^28^ | **L** | **L** | **L** | **L** | **L** | **L** | **L** |
| Foglia 2017 ^29^ | **L** | **L** | **L** | **L** | **L** | **L** | **L** |
| Ebmeier 2018 ^29^ | **L** | **L** | **L** | **L** | **L** | **L** | **L** |
| Harris 2019 ^30^ | **L** | **L** | **L** | **H** | **L** | **L** | **L** |
| Pilcher 2020 ^31^ | **L** | **L** | **L** | **L** | **L** | **L** | **L** |
| Harskamp 2021^32^ | **L** | **L** | **L** | **L** | **L** | **L** | **L** |
| Nguyen2022 ^33^ | **L** | **L** | **L** | **L** | **L** | **L** | **L** |
| Sjoding 2020 ^34^ | **L** | **L** | **L** | **H** | **L** | **L** | **L** |
| Wong 2021 ^35^ | **L** | **L** | **L** | **H** | **L** | **L** | **L** |
| Valbuena 2022 ^36^ | **L** | **L** | **L** | **H** | **L** | **L** | **L** |
| Valbuena 2022 ^37^ | **L** | **L** | **L** | **?** | **L** | **L** | **L** |
| Fawzy 2022 ^38^ | **L** | **L** | **L** | **H** | **L** | **L** | **L** |
| Henry 2022 ^39^ | **L** | **L** | **L** | **L** | **L** | **L** | **L** |
| Sudat 2022 ^40^ | **L** | **L** | **L** | **H** | **L** | **L** | **L** |
| Bangash 2022 ^40^ | **L** | **L** | **L** | **H** | **L** | **L** | **L** |
| Wiles 2022 ^9^ | **L** | **L** | **L** | **L** | **L** | **L** | **L** |
| Wiles 2022 ^41^ | **L** | **L** | **L** | **L** | **L** | **L** | **L** |
| Burnett 2022 ^42^ | **L** | **L** | **L** | **L** | **L** | **L** | **L** |
| Andrist 2022 ^43^ | **L** | **L** | **L** | **H** | **L** | **L** | **L** |
| Chesley 2022 ^44^ | **L** | **L** | **L** | **H** | **L** | **L** | **L** |
| Savorgnan 2022 ^45^ | **L** | **L** | **L** | **?** | **L** | **L** | **L** |
| Seitz 2022 ^46^ | **L** | **L** | **L** | **H** | **L** | **L** | **L** |
| Crooks 2022 ^47^ | **L** | **L** | **L** | **H** | **L** | **L** | **L** |
| Vesoulis 2022 ^26^ | **L** | **L** | **L** | **H** | **L** | **L** | **L** |
| Pritchett 2022 ^48^ | **L** | **L** | **L** | **?** | **L** | **L** | **L** |

**L** = Low risk of bias

**H** = High risk of bias

**?** = Risk of bias unclear

## **Supplementary table 2.** Prospective healthy volunteer studies included in the review (n=8)

| **Article** | **Study design** | **Geographical setting** | **Study size** | **Paired samples** | **Make(s) of pulse oximeter** | **Skin tone assessment** | **Proportion of each skin tone category** | **Skin tone / ethnicity related findings** |
| --- | --- | --- | --- | --- | --- | --- | --- | --- |
| Saunders  1976^25^ | Healthy adults exposed to hypoxia | America | 24 | 223 | HP (multi-wave) 47201A (ear probe) | None | 5 (21%) Asian or African participants | No inaccuracy detected.  Good correlation between SaO_2_ and SpO_2_. |
| Mendelson  1988^26^ | Healthy adults exposed to hypoxia | America | 15 | 135 | Datascope ACCUSAT, and HP 47201A ear probe oximeter | None | 3 Black (20%), 2 Oriental (13%) and 10 (67%) White participants | No inaccuracy detected.  Good correlation between HP SaO_2_ and Datascope SpO_2_ in Black participants |
| Zeballos  1991^36^ | Healthy exercising adults exposed to hypoxia | America | 33 | 521 | HP 47201A (ear probe), Biox Ohmeda IIA (ear probe) | None | 33 Black (100%) participants | Over-estimation (bias) of SaO_2_ with the Biox (but not HP) which was greater with hypoxia |
| Bickler  2005^26^ | Healthy adults exposed to hypoxia | America | 21 | 1067 | Nellcor N-595 with OxiMax-A probe, Novametrix 513 and Nonin Onyx | None | 11 (52%) with dark and 10 (48%) with light skin tones | Over-estimation (bias) of SaO_2_ in participants with dark skin tones, which was greater when as hypoxaemia increased |
| Feiner  2007^28^ | Healthy adults exposed to hypoxia | America | 36 | / | Masimo Radical, Nellcor N-595, Nonin 9700 | Subjective assessment of photographs | 17 (47%) dark, 7 (19%) intermediate, and 12 (33%) light skin tone | Over-estimation (bias) of SaO_2_ in dark skin participants, worse when hypoxic |
| Barker  2022^28^ | Retrospective combining of healthy volunteer data (exposed to hypoxia) | / | 75 | 7183 | Masimo SET pulse oximeters with RD SET sensors | None | 39 (52%) Black and 36 (48%) White participants | The bias, precision and accuracy for Black participants was -0.20%, 1.40 and 1.42 respectively; for White participants it was -0.05%, 1.35 and 1.35 respectively. The bias difference between the White and Black subgroups was 0.15 (p-value < 0.001). |
| Gudelunas  2022^29^ | Healthy adults exposed to hypoxia | America | 146 | 9763 | Nellcor N-595 and Masimo Radical 7 | Fitzpatrick scale | 25 (17%) with light skin (Fitzpatrick class I-II), 78 (53%) with medium (class III-IV), and 43 (29%) with dark (class V-VI) skin | In a multivariable mixed-effects model incorporating repeated-measures and different levels of SaO_2_ and perfusion, skin pigment, perfusion index and degree of hypoxemia significantly contributed to bias in both pulse oximeters. |
| Ajizian  2023^29^ | Retrospective analysis of healthy volunteer data** | / | 88 | / | Nellcor (MAXA, MAXN, MAXFAST, or FLEXMAX) | Visual 4-level scale: very light, olive, dark olive, and extremely dark | 69 (78%) very light / olive, 19 (22%) dark olive / extremely dark | Increased errors in participants with dark skin tones. For SaO_2_ of 80-92%, the light and dark skin pigmentation groups had bias of 0.05% (95%CI 0.01-0.09) and 0.30% (95%CI 0.22-0.38); precision of 1.39% (95%CI 1.36-1.41) and 1.54% (95%CI 1.49-1.60); and root-mean square deviation of 1.39% and 1.57%. |

HP = Hewlett-Packard

* funded by Masimo ** funded by Medtronic

## **Supplementary table 3.** Prospective clinical studies included in the review (n=18)

| **Article** | **Study design** | **Geographical setting** | **Study size** | **Paired samples** | **Make(s) of pulse oximeter** | **Skin tone measurement** | **Proportion of each skin tone category** | **Skin tone / ethnicity related findings** |
| --- | --- | --- | --- | --- | --- | --- | --- | --- |
| Wang  1985 ^30^ | Adult hospital patients (in and outpatients) | Singapore | 31 | / | Ohmeda Biox III (ear and finger probes) | None | 22 (71%) Chinese, 6 (19%) Malay, 3 (10%) Indian | No inaccuracy detected.  Good correlation between SaO_2_ and SpO_2_ |
| Ries  1985 ^31^ | Adult respiratory medicine outpatients undertaking an exercise test | America | 23 | 99 | HP 47201A (ear), Biox IIA (ear probe) | Subjective semi-quantitative scale (1-4) | 5 (22%) moderate and 18 (78%) light pigmentation | The differences between SpO_2_ and SaO_2_ were greater in darker skin tone group with HP but not Biox device (values compared by t-test). |
| Emery  1987 ^32^ | Infants in a neonatal ICU | America | 9 | / | Nellcor N-100 | Subjective assessment (light / medium / dark) | 9 (100%) Black | Over-estimation (bias) of SaO_2_ (2.4 ± 2.4%) by SpO_2_ |
| Gabrielczyk  1988 ^37^ | Mechanically ventilated adults post cardiac surgery | America | 21 | 68 | Nellcor N-100 | None | 4 (19%) with pigmented skin and 17 (81%) with non-pigmented skin | No inaccuracy detected.  Good correlation between SaO_2_ and SpO_2_ |
| Cecil  1988 ^33^ | Hospitalised adult patients | America | 152 | 335 | Nellcor N-100, Ohmeda 3700 | Visual scale, 0-3 (light to dark) | 1 (0.7%) Asian and 15 (11%) Black and 136 (89%) White participants | Correlation demonstrated greater inaccuracy in Black versus White participants (over-estimation of SaO_2_ for Biox, under-estimation of SaO_2_ for HP) |
| Ries  1989 ^39^ | Adult respiratory medicine outpatients undertaking an exercise test | America | 187 | 1000 | Biox III (ear), HP 47201A (ear) | Munsell skin colour assessment (graded 1-4) | 13% grade 1; 50% grade 2; 22% grade 3; 15% grade 4 | More errors and greater inaccuracy in the darkest skin tone group (grade 4). The differences between the mean SpO_2_ and SaO_2_ were approximately 0.5% higher in the two darker skin tone groups compared to the two lighter groups |
| Escourrou  1990 ^34^ | Adult respiratory medicine outpatients undertaking an exercise test | France | 101 | 195 | Ohmeda Biox 3700, Criticare CSI 501+, Nellcor N200 | None | 5 (5%) moderately pigmented and 96 (95%) non-pigmented skin | No inaccuracy detected.  Good correlation between SaO_2_ and SpO_2_ |
| Lee  1993 ^41^ | Critically ill adults on ICU | Singapore | 33 | 150 | Nellcor, Simed, and Critikon | None | 22 (67%) Chinese, 6 (18%) Malay, 5 (15%) Indian | Pulse oximetry overestimation of SaO_2_ (bias) increased with darker skin tone (Indian > Malay > Chinese; approximately 1.7±0.7% for Indian participants) |
| Bothma  1996 ^35^ | Critically ill adults on ICU | South Africa | 100 | / | Simed S100e, Nihon Koden, Ohmeda 3740 (ear and finger) | Reflectance spectrophotometry | (100) 100% darkly pigmented | No inaccuracy (difference in bias) detected. |
| Avant  1997 ^33^ | Critically ill children on ICU | America | 50 |  | Nellcor Oxiband and Dura-Y (digit wraps) | None | 15 (30%) Black and 35 (70%) White participants | No inaccuracy (difference in bias) detected. |
| Adler  1998 ^36^ | Adult patients admitted to an ED | America | 295 |  | Nellcor D-25 | Munsell colour tile system + a scale of light, intermediate and dark skin tone | 51% light, 37% intermediate, 12% dark skin tone | No inaccuracy (difference in bias) detected, however, pulse oximetry functioned poorly more often in those with dark skin |
| Ross  2014 ^35^ | Hypoxaemic children on paediatric ICUs | America | 225 | 1980 | Masimo poximeter + LCNS probes and Nellcor pulse oximeters | None | 20 (9%) African American, 94 (42%) Hispanic, 7 (3%) Asian, 94 (42%) White [10 (4%) other] | Lower likelihood of bias in African American children |
| Foglia  2017 ^37^ | Hospitalised cyanotic children with SpO2 <90% | America | 35 | / | Nellcor Oximax + GE Solar 8000, Masimo Rainbow SET Radical 7 | Munsell colour chart | 14 (39%) dark, 21 (58%) light skin | No inaccuracy detected.  No statistical difference in bias reported (3.0 ± 5.0% for light tone and 5.4 ± 5.1% for dark skin tone, p=0.39). |
| Ebmeier  2018 ^37^ | Critically ill adults on ICU | New Zealandand Australia | 394 | / | Marquette Rac-4A monitors + Masimo sensors, Philips IntelliVue MP70 monitors + Philips reusable sensors | Fitzpatrick scale: then light (1 or 2), medium (3 or 4), or dark (5 or 6) | 80.6% European, 7.6% Asian, 7.1% Māori, 2.4% Pacific Island [2.4% other] | Dark skin tone associated with greater bias (over-estimation of SaO_2_ in light vs dark and light vs medium skin tones) |
| Harris  2019 ^38^ | Infant patients with cyanotic heart disease on ICU | America | 24 | 185 | Masimo LCNS saturation sensor + Philips monitor, WristOx2 3150 + infant sensors 8008J | Massey skin colour score | 1 (4%) Asian, 2 (8%) African American, 3 (13%) Native Hawaiian or Pacific Islander and 16 (67%) White participants | No inaccuracy (difference in bias) detected. |
| Pilcher  2020 ^31^ | Adult patients (outpatient, on wards or HDUs) | Australis and New Zealand | 400 | 400 | Nonin, Masimo and other (see below)* | Fitzpatrick scale | Fitzpatrick score of  I: 44 (11%),  II: 198 (50%), III: 127 (32%), IV: 30 (8%),  V: 1 (0.3%),  VI: 0 (0%) | No inaccuracy detected.  Using an ANOVA, there was no difference in bias between light (I+II) and medium (III+IV) skin tone groups. |
| Harskamp  2021^36^ | Adult patients on ICU | The Netherlands | 35 | 234 | FAC FS10D, AGPTEK FS10C, ANAPULSE ANP 100, Cocobear, Contec CMS50D1, HYLOGY MD-H37, Mommed YM101, PRCMISEMED F4PRO, PULOX PO-200, Zacurate Pro Series 500DL | Fitzpatrick scale | 5 (14%) with Fitzpatrick grade VI-VI, 30 (86%) with Fitzpatrick grade I-IV | Increased bias related to darker skin tone (I-III vs IV-VI) in 5 of the 10 pulse oximeters |
| Nguyen  2022 ^40^ | Adult patients with ARDS on an ICU (COVID-19 and non-COVID-19) | France | 55 |  | Masimo ear probe | None | 17 (31%) Black and 38 (69%) White participants | Comparison of correlations showed Black ethnicity was associated with a greater over-estimation of SaO_2_ |

* Nonin (Avant 9700, Avant 4000, Avant unspecified, Lifesense Medair, 2120, 2140); Masimo (Masimoset Quartz Q400, Masimoset Quartz, unspecified*, Rainbow Radical); Novametrix Model 512, GE Dash 3000; Welch Allyn monitor with a Nellcor probe; Philips Intellivue MP70 monitor with a GE TruSignal, Nellcor or Philips probe; Ohmeda Biox 3700E monitor with a GE TruSignal or Nellcor probe; Carescape Monitor B450 with a Nellcor probe.

ABG = arterial blood gas; co-ox = co-oximetry; ICU = intensive care unit; ED = emergency department; ARDS = acute respiratory distress syndrome; HDU = high dependency unit

## **Supplementary table 4.** Retrospective clinical studies included in the review (n=18)

| **Article** | **Study design** | **Geographical setting** | **Study size** | **Paired samples** | **Make(s) of pulse oximeter** | **Skin tone measurement** | **Proportion of each ethnicity (self-reported)** | **Skin tone / ethnicity related findings** |
| --- | --- | --- | --- | --- | --- | --- | --- | --- |
| Sjoding  2020 ^38^ | Two data sets:  i) adult inpatients receiving supplemental oxygen ii) adult ICU patients. | America | i) 1609  ii) 8392 | i) 10,780  ii) 37,308 | / | None | i) 276 (17%) Black and 1333 (83%) White  ii) 1050 (13%) Black and 7342 (87%) White | i) OH in 11.7 (8.5-16.0) % of Black and 3.6 (2.7-4.7) % of White patients (with an SpO_2_ of >92%).  ii) OH in 17.0 (12.2 to 23.3) % of Black and 6.2 (5.4 to 7.1) % of White patients (with an SpO_2_ of >92%). |
| Wong  2021 ^39^ | Combination of data from five adult ICU databases | America | 79,044 | 87,971 | / | None | 1919 (2.3%) Asian; 26,032 (29.6%) Black, 2397 (2.7%) Hispanic and 57,632 (65.5%) White | OH in 6.9 % of Black, 6.0 % of Hispanic, 4.9 % of Asian and 4.9 % of White patients (P < 0.01). |
| Valbuena  2022 ^40^ | Adult general surgical and medical patients | America | / | 30,039 | / | None | Paired samples: Black 6498 (22%), Hispanic or Latino 1623 (5%), White 21,918 (73%) | OH in 19.6 (18.6-20.6) % of Black, 16.2 (14.4-18.1) % of Hispanic or Latino and 15.6 (15.0-16.1) % of White patients (p<0.001) (with an SpO_2_ of >92%).  Greater bias and less precision in Black patients. |
| Valbuena 2022 ^41^ | Adult ICU patients with ARDS or COVID-19 about to receive ECMO | America | 372 | 1562 | / | None | 65 (17%) Asian, 51 (14%) Black, 70 (19%) Hispanic, 186 (50%) White | OH in 10.2 (6.2%-15.3) % of White and 21.5% (11.3%-35.3% of Black patients (P =0.031); OH in 8.6% (3.2%-17.7) % of Hispanic patients (P = 0.693 vs White); OH in 9.2% (3.5%-19.0) % of Asian patients (P =0.820 vs White). For all patients in whom pre-ECMO SpO_2_ measurements were between 92% and 96%. |
| Fawzy  2022 ^41^ | Adult hospitalised patients with COVID-19 from 5 centres | America | 1216 | 32,282 | / | None | 63 (5%) Asian, 478 (39%) Black, 215 (18%) Hispanic, 420 (38%) White | OH in 30.2 % of Asian, 28.5 % of Black, 29.8 % of Hispanic and 17.2. % of White patients.  Over-estimation of SaO_2_ was 1.7 (0.5%-3.0) % in Asian, 1.2 (0.6%-1.9) % in Black and 1.1 (0.3%-1.9) % in Hispanic patients. Delay of 1.0 (0.23-1.9; p=0.01) hours in receiving oxygen amongst Black patients. |
| Henry  2022 ^42^ | Adults admitted to ICU or undergoing surgery during inpatient hospitalisation | America | 26,603 | 128,285 | / | None | 273 (1%) Indigenous American, 574 (2%) Asian, 1263 (5% Black and 24,493 92%) White | Accounting for multiple observations, OH had an estimated probability of 6.2 (5.1–7.6) % in Black patients, 6.6 (4.9–8.8) % in Asian patients, and 6.6 (4.4–10.0) % in American Indian patients, compared to 3.6 (3.4–3.8) % in White patients. |
| Sudat  2022 ^42^ | Two data sets:  a) hospitalised adults with COVID-19 presenting to the ED; b) adults with COVID-19 presenting to the ED | America | i) 13,130  ii) 8735 | i) 43,753  ii) 8735 | / | None | i) Participants: 2616 (20%) Black and 10,514 (80%) White  ii) 1699 (19%) Black and 7036 (81%) White | i) Overall: mean SpO_2_-SaO_2_ difference in Black patients was 2.45 % and in White patients 1.53 %. OH in Black patients was 5.50 % and 3.01 % in White patients (P<0.001) ii) SpO_2_-SaO_2_ difference 1 % higher in Black patients which was associated with lower admission probability, dexamethasone treatment and oxygen therapy. |
| Bangash  2022 ^43^ | Adults admitted to four hospitals | United Kingdom | 16,818 | 16,818 | / | None | Of the paired measurements: 1965 (12%) Asian, 674 (4% Black), 13,649 (81%) White | Mean SpO_2_-SaO_2_ difference in Black patients was 0.8 (-0.3-1.9) % and in White patients 0.4 (-1.2-1.4) %.  OH in Black patients was 8.7 % and 6.1 % in White patients (p=0.012)  Specificity (the probability of SpO_2_ being low when SaO_2_ is low):  White specificity = 68.5% (66.5-70.5%)  Black = 51.4 (41.5-61.3%)  Asian = 58.9 (53.2-64.5)% |
| Wiles  2022 ^43^ | Adult ICU patients with COVID receiving non-invasive ventilation | United Kingdom | 194 | 6,216 | / | None | 34 (18%) Asian, 19 (10%) Black and 135 (70%) White [6 other] | No inaccuracy detected.  Bias was 0.28% (1.79–2.35), 0.33% (2.47–2.35) and  0.75% (3.47–1.97) for White, Asian and Black patients respectively.  The incidence of SpO_2_ measurements being >90% with the paired SaO_2_ value being ≤90% was 5.7% (95%CI 5.0–6.4) for White, 4.9% (95%CI 3.9–6.3) for Asian and 6.3% (95%CI 4.7–8.6) for Black patients.  In total, 534/16,438 (3.2%) paired readings from White patients had showed OH, 100/1875 (5.3%) paired readings from Black patients showed OH |
| Wiles  2022 ^44^ | Retrospective analysis of mechanically ventilated adult patients with COVID-19 from a single centre | United Kingdom | 178 | 24,626 | B1x5M/P monitoring system and Nellcor or disposable (Mindray)probes | None | 30 South Asian, 13 Black, 126 White and 9 other | Accuracy [√ (bias^2^+precison^2^)] was 2.30% for White, 2.56% for South Asian and 3.07% for Black patients.  OH detected in 5.33 (4.32 6.35) % of Black, 2.90 (2.43 – 3.36) % of South Asian and 3.25 (2.98 – 3.52) % of White patients. |
| Burnett  2022  ^45^ | Patients undergoing anaesthesia at one centre | America | 46,253 | 151,070 | Nellcor and Masimo | None | 2612 (6%) Asian, 6304 (14%) Hispanic, 5177 (11%) Black, and 22,089 (48%) White [10,071 (22%) other] | OH detected in 2.1% of paired readings in Black patients and 1.8% of paired readings in Hispanic patients versus 1.1% of paired readings in White patients (P < 0.001 for both).  The frequency of OH at a patient level (at any point) was:   - 3.3% for White patients - 6.2% for Black patients - 3.3% for Asian patients - 5.5% for Hispanic patients   SpO_2_ bias % (mean ± standard deviation) was: +0.2 ± 6.5% for Asian, +0.6 ± 9.1% for Black, +0.5 ± 7.9% for Hispanic and −0.2 ± 6.3% for White patients. |
| Andrist  2022 ^46^ | Hospitalised children (aged 17 years or younger) | America | 1061 | 9023 | / | None | 183 (17%) Black and 878 (83%) White | Mean (SD) SpO_2_ bias was 4.3 (5.0) % in Black and 3.5 (5.0) % in White and patients.  The frequency of OH was 9.6 (6.3% - 14.5) % of paired readings among Black patients and 5.8 (4.6% -7.3) % of paired readings among White patients.  At the patient level, 134 of 860 White patients (15.6%; 95% CI, 13.3%-18.2%) and 38 of 180 Black patients (21.1%; 95% CI, 15.7%-27.7%) had occult hypoxemia episodes. |
| Chesley  2022 ^40^ | Adult critically ill patients on ICUs at two centres | America | 7693 | 105,467 | / | None | 239 (3%) Asian / Pacific Islander, 1919 (25%) Black, 17 (0.2%) Indigenous, 226 (3%) Latinx, and 4621 (60%) White [220 (3%) other] | Frequency of OH was 7.9 % of paired readings in Black patients and 2.9% of paired readings in White patients (P < .001).  11.0% of White patients had OH at some point and 22.0% of Black patients.  In an adjusted model, the odds ratio for OH among SpO_2_ measurements for Black patients was 2.16 (95% CI, 1.36-3.44) % compared with White patients. |
| Savorgnan  2022 ^41^ | Retrospective study of children admitted to a single centre with COVID | America | 4300 | / | / | None | / | Black ethnicity was associated with worse pulse oximeter bias (OR -1.94 (-3.22 to - 0.65), p<0.003) [overestimation, worse with increasing hypoxaemia] |
| Seitz  2022  ^43^ | Adult patients in an ICU (excluding COVID) at one centre | America | 1024 | 5557 | / | None | Of the paired measurements: 769 (14%) Black and 4788 (86%) White | Among patients with a SpO_2_ value between 92% - 96%, Black patients were more likely to have both hypoxaemia (OH) (3.5 vs 1.1 %; p = 0.002) and hyperoxaemia (4.7 vs 2.4 %; p = 0.03), compared with White patients.  Across the range of SpO_2_ values of 92 – 98%, the associated SaO_2_ value was approximately 1 percentage point lower for Black patients compared with White patients. |
| Crooks  2022  ^42^ | Adult hospitalised patients with COVID-19 from a single centre | United Kingdom | 2997 | 5374 | / | None | Of the paired measurements: 246 (5%) Asian, 151 (3%) Black, 3946 (73%) White, 6 (0.1%) mixed | SpO_2_ over-estimated SaO_2_ by 6.9 (−21.9 to +35.8) % in Mixed, 5.4 (−25.9 to +36.8) % in Black, 5.1 (−23.8 to 34.0) % in Asian and 3.2% (−22.8 to +29.1) % in White patients. These differences were particularly marked in the clinically important range of 85% to 89% with mean SpO_2_ values almost 5% higher than SaO_2_ in Black, Asian or mixed ethnicity |
| Vesoulis  2022  ^44^ | Premature infants (<32 weeks gestation) on ICU | America | 294 | 4387 | Nellcor SpO_2_ module + Philips IntelliVue MP70, MX800 + Neonatal-Adult MAX-N adhesive sensors | None | 124 (42%) Black, 170 (58%) White | SaO_2_ overestimation (mean bias) 2.4 fold greater for Black infants.  OH detected in 9.2 % of Black and 7.7% of White infants (NS).  SpO_2_ to detect hypoxia: sensitivity and specificity were similar for Black infants (39% sensitive, 81% specific) and White infants (38% sensitive, 78% specific). |
| Pritchett  2022  ^45^ | Children with congenital cyanotic heart disease undergoing cardiac catheterisation | America | 123 | 425 | / | None | 29 (24%) African American | In African American children, SpO_2_ increasingly overestimated SaO_2_ at lower SaO_2_. At an SaO_2_ of 80% the expected SpO_2_ for African American patients was 82% versus 81% for non-African American patients |

ABG = arterial blood gas; co-ox = co-oximetry; ICU = intensive care unit; ED = emergency department; ARDS = acute respiratory distress syndrome; OH = occult hypoxaemia; SD = standard deviation; NS = non-significant

**Supplementary table 5**. The definition and incidence of occult hypoxaemia in retrospective clinical studies

| **Article** | **Definition of occult hypoxaemia** |
| --- | --- |
| Sjoding  2020 | SaO_2_ < 88% with a paired SpO_2_ 92-96% (or ≥92%) |
| Wong  2021 ^35^ | SaO_2_ < 88% with a paired SpO_2_ ≥ 88% |
| Valbuena  2022 ^36^ | SaO_2_ < 88% with a paired SpO_2_ 92-96% (or 92%) |
| Valbuena  2022 ^37^ | SaO_2_ ≤ 88% with a paired SpO_2_ 92-96% |
| Fawzy  2022 ^38^ | SaO_2_ < 88% with a paired SpO_2_ 92-96% (or ≥ 92%) |
| Henry  2022 ^39^ | SaO_2_ < 88% with a paired SpO_2_ of 92-96% (or ≥92%) |
| Sudat  2022 ^40^ | Hypoxaemia defined as SaO_2_ < 90% |
| Bangash  2022 ^7^ | Hypoxaemia defined as SaO_2_ < 94% |
| Wiles  2022 ^9^ | Hypoxaemia defined as SaO_2_ < 90% |
| Wiles  2022 ^41^ | SaO_2_ ≤ 90% with a paired SpO_2_ ≥ 92% |
| Burnett  2022 ^42^ | SaO_2_ < 88% with a paired SpO_2_ 92-96% (or ≥ 92%) |
| Andrist  2022 ^43^ | SaO_2_ < 88% with a paired SpO_2_ 92-96% (or ≥ 92%) |
| Chesley  2022 ^44^ | SaO_2_ < 88% with a paired SpO_2_ 92-96% |
| Seitz  2022 ^46^ | SaO_2_ < 88% with a paired SpO_2_ 92-96% |
| Vesoulis  2022  ^8^ | SaO_2_ < 85% with a paired SpO_2_ > 90% |

* P<0.05 or 95% CIs do not overlap

OH = Occult hypoxaemia

**References**

1. Ochoa-Gutierrez V, Guerrero-Zuñiga S, Reboud J, Pazmino-Betancourth M, Harvey AR, Cooper JM. Changes in Oxygenation Levels During Moderate Altitude Simulation (Hypoxia-Induced): A Pilot Study Investigating the Impact of Skin Pigmentation in Pulse Oximetry. *Adv Exp Med Biol*; 2022; **1395**: 391–6

2. Chapman KR, D’Urzo A, Rebuck AS. The accuracy and response characteristics of a simplified ear oximeter. *Chest*; 1983; **83**: 860–4

3. Cahan C, Decker MJ, Hoekje PL, Strohl KP. Agreement between noninvasive oximetric values for oxygen saturation. *Chest*; 1990; **97**: 814–9

4. Avant MG, Lowe N, Torres A Jr. Comparison of accuracy and signal consistency of two reusable pulse oximeter probes in critically ill children. *Respiratory Care* 1997; **42**: 698–704

5. Adler JN, Hughes LA, Vivilecchia R, Camargo CA Jr. Effect of skin pigmentation on pulse oximetry accuracy in the emergency department. *Acad Emerg Med* 1998; **5**: 965–70

6. Foglia EE, Whyte RK, Chaudhary A, et al. The Effect of Skin Pigmentation on the Accuracy of Pulse Oximetry in Infants with Hypoxemia. *J Pediatr* 2017; **182**: 375-377.e2

7. Bangash MN, Hodson J, Evison F, et al. Impact of ethnicity on the accuracy of measurements of oxygen saturations: A retrospective observational cohort study. *EClinicalMedicine* 2022; **48**: 101428

8. Vesoulis Z, Tims A, Lodhi H, Lalos N, Whitehead H. Racial discrepancy in pulse oximeter accuracy in preterm infants. *J Perinatol* 2022; **42**: 79–85

9. Wiles MD, El-Nayal A, Elton G, et al. The effect of patient ethnicity on the accuracy of peripheral pulse oximetry in patients with COVID-19 pneumonitis: a single-centre, retrospective analysis. *Anaesthesia* 2022; **77**: 143–52

10. Lee J, Kim KW, Choi SH, Huh J, Park SH. Systematic Review and Meta-Analysis of Studies Evaluating Diagnostic Test Accuracy: A Practical Review for Clinical Researchers-Part II. Statistical Methods of Meta-Analysis. *Korean J Radiol* 2015; **16**: 1188–96

11. Saunders NA, Powles AC, Rebuck AS. Ear oximetry: accuracy and practicability in the assessment of arterial oxygenation. *Am Rev Respir Dis* 1976; **113**: 745–9

12. Mendelson Y, Kent JC, Shahnarian A, Welch GW, Giasi RM. Evaluation of the Datascope ACCUSAT pulse oximeter in healthy adults. *J Clin Monit* 1988; **4**: 59–63

13. Zeballos RJ, Weisman IM. Reliability of noninvasive oximetry in black subjects during exercise and hypoxia. *Am Rev Respir Dis* 1991; **144**: 1240–4

14. Bickler PE, Feiner JR, Severinghaus JW. Effects of skin pigmentation on pulse oximeter accuracy at low saturation. *Anesthesiology* 2005; **102**: 715–9

15. Feiner JR, Severinghaus JW, Bickler PE. Dark skin decreases the accuracy of pulse oximeters at low oxygen saturation: the effects of oximeter probe type and gender. *Anesth Analg* 2007; **105**: S18–23

16. Barker SJ, Wilson WC. Accuracy of Masimo SET pulse oximetry in black and white volunteer subjects: a retrospective review. Abstracts of Papers Presented at the 2022 Virtual Annual Meeting of the Society for Technology in Anesthesia (STA) January 13-15, 2022. *Anesth Analg* 2022; **134**: 1–81

17. Gudelunas MK, Lipnick MS, Hendrickson CM, et al. Low perfusion and missed diagnosis of hypoxemia by pulse oximetry in darkly pigmented skin: A prospective study. *medRxiv* 2022; 2022–2010

18. Ajizian S, McGonigle S, Dove J, Ting Y-J, Sethi R, Milkes D. 1030: Occult hypoxemia and pulse oximetry performance across skin pigmentation groups. *Crit Care Med* 2023; **51**: 509

19. Wang YT, Poh SC. Noninvasive oximetry in pigmented patients. *Ann Acad Med Singapore* 1985; **14**: 427–9

20. Ries AL, Farrow JT, Clausen JL. Accuracy of two ear oximeters at rest and during exercise in pulmonary patients. *Am Rev Respir Dis* 1985; **132**: 685–9

21. Emery JR. Skin pigmentation as an influence on the accuracy of pulse oximetry. *J Perinatol* 1987; **7**: 329–30

22. Gabrielczyk MR, Buist RJ. Pulse oximetry and postoperative hypothermia. An evaluation of the Nellcor N-100 in a cardiac surgical intensive care unit. *Anaesthesia* 1988; **43**: 402–4

23. Cecil WT, Thorpe KJ, Fibuch EE, Tuohy GF. A clinical evaluation of the accuracy of the Nellcor N-100 and Ohmeda 3700 pulse oximeters. *J Clin Monit Comput* Springer Science and Business Media LLC; 1988; **4**: 31–6

24. Ries AL, Prewitt LM, Johnson JJ. Skin color and ear oximetry. *Chest* 1989; **96**: 287–90

25. Escourrou PJ, Delaperche MF, Visseaux A. Reliability of pulse oximetry during exercise in pulmonary patients. *Chest* Elsevier; 1990; **97**: 635–8

26. Lee KH, Hui KP, Tan WC, Lim TK. Factors influencing pulse oximetry as compared to functional arterial saturation in multi-ethnic Singapore. *Singapore Med J* 1993; **34**: 385–7

27. Bothma PA, Joynt GM, Lipman J, et al. Accuracy of pulse oximetry in pigmented patients. *S Afr Med J* 1996; **86**: 594–6

28. Ross PA, Newth CJL, Khemani RG. Accuracy of pulse oximetry in children. *Pediatrics* 2014; **133**: 22–9

29. Ebmeier SJ, Barker M, Bacon M, et al. A two centre observational study of simultaneous pulse oximetry and arterial oxygen saturation recordings in intensive care unit patients. *Anaesth Intensive Care* 2018; **46**: 297–303

30. Harris BU, Stewart S, Verma A, et al. Accuracy of a portable pulse oximeter in monitoring hypoxemic infants with cyanotic heart disease. *Cardiol Young* 2019; **29**: 1025–9

31. Pilcher J, Ploen L, McKinstry S, et al. A multicentre prospective observational study comparing arterial blood gas values to those obtained by pulse oximeters used in adult patients attending Australian and New Zealand hospitals. *BMC Pulm Med* 2020; **20**: 1–9

32. Harskamp RE, Bekker L, Himmelreich JCL, et al. Performance of popular pulse oximeters compared with simultaneous arterial oxygen saturation or clinical-grade pulse oximetry: a cross-sectional validation study in intensive care patients. *BMJ open respiratory research* Archives of Disease in childhood; 2021; **8**: e000939

33. Nguyen LS, Helias M, Raia L, et al. Impact of COVID-19 on the association between pulse oximetry and arterial oxygenation in patients with acute respiratory distress syndrome. *Sci Rep* 2022; **12**: 1462

34. Sjoding MW, Dickson RP, Iwashyna TJ, Gay SE, Valley TS. Racial Bias in Pulse Oximetry Measurement. *N Engl J Med* 2020; **383**: 2477–8

35. Wong A-KI, Charpignon M, Kim H, et al. Analysis of Discrepancies Between Pulse Oximetry and Arterial Oxygen Saturation Measurements by Race and Ethnicity and Association With Organ Dysfunction and Mortality. *JAMA Netw Open* 2021; **4**: e2131674

36. Valbuena VSM, Seelye S, Sjoding MW, et al. Racial bias and reproducibility in pulse oximetry among medical and surgical inpatients in general care in the Veterans Health Administration 2013-19: multicenter, retrospective cohort study. *BMJ* 2022; **378**: e069775

37. Valbuena VSM, Barbaro RP, Claar D, et al. Racial Bias in Pulse Oximetry Measurement Among Patients About to Undergo Extracorporeal Membrane Oxygenation in 2019-2020: A Retrospective Cohort Study. *Chest* 2022; **161**: 971–8

38. Fawzy A, Wu TD, Wang K, et al. Racial and Ethnic Discrepancy in Pulse Oximetry and Delayed Identification of Treatment Eligibility Among Patients With COVID-19. *JAMA Intern Med* 2022; **182**: 730–8

39. Henry NR, Hanson AC, Schulte PJ, et al. Disparities in Hypoxemia Detection by Pulse Oximetry Across Self-Identified Racial Groups and Associations With Clinical Outcomes. *Crit Care Med* 2022; **50**: 204–11

40. Sudat SEK, Wesson P, Rhoads KF, et al. Racial Disparities in Pulse Oximeter Device Inaccuracy and Estimated Clinical Impact on COVID-19 Treatment Course. *Am J Epidemiol* 2023; **192**: 703–13

41. Wiles MD, El-Nayal A, Elton G, et al. Effect of patient ethnicity on the accuracy of peripheral pulse oximetry in patients with COVID-19 pneumonitis requiring mechanical ventilation. *Anaesthesia* 2022; **77**: 489–91

42. Burnett GW, Stannard B, Wax DB, et al. Self-reported Race/Ethnicity and Intraoperative Occult Hypoxemia: A Retrospective Cohort Study. *Anesthesiology* 2022; **136**: 688–96

43. Andrist E, Nuppnau M, Barbaro RP, Valley TS, Sjoding MW. Association of Race With Pulse Oximetry Accuracy in Hospitalized Children. *JAMA Netw Open* 2022; **5**: e224584

44. Chesley CF, Lane-Fall MB, Panchanadam V, et al. Racial disparities in occult hypoxemia and clinically based mitigation strategies to apply in advance of technological advancements. *Respir Care* 2022; **67**: 1499–507

45. Savorgnan F, Acosta S, Borges N. Racial Bias in Pulse Oximetric in Pediatric COVID Patients. *Circulation* 2022; **146**: A14058–A14058

46. Seitz KP, Wang L, Casey JD, et al. Pulse Oximetry and Race in Critically Ill Adults. *Crit Care Explor* 2022; **4**: e0758

47. Crooks CJ, West J, Morling JR, et al. Pulse oximeter measurements vary across ethnic groups: an observational study in patients with COVID-19. *Eur Respir J* [Internet] 2022; **59** Available from: http://dx.doi.org/10.1183/13993003.03246-2021

48. Pritchett CM, O’Halloran CP, Lay AS, Monahan A, Tannous P. Pulse Oximetry Error in Patients with Single Ventricle Palliation Increases as a Function of Systemic Hypoxemia and Patient Race. *Pediatrics* 2022; **149**: 367–367
